# Legal regulation of AI-assisted academic writing: challenges, frameworks, and pathways

**DOI:** 10.3389/frai.2025.1546064

**Published:** 2025-04-07

**Authors:** Runyang Gao, Danghui Yu, Biao Gao, Heng Hua, Zhaoyang Hui, Jingquan Gao, Cha Yin

**Affiliations:** ^1^Faculty of Law and Justice, University of New South Wales, Sydney, NSW, Australia; ^2^Teaching and Research Support Center, Naval Medical University, Shanghai, China

**Keywords:** artificial intelligence, academic writing, copyright protection, academic integrity, data security, legal regulation

## Abstract

**Introduction:**

The widespread application of artificial intelligence in academic writing has triggered a series of pressing legal challenges.

**Methods:**

This study systematically examines critical issues, including copyright protection, academic integrity, and comparative research methods. We establishes a risk assessment matrix to quantitatively analyze various risks in AI-assisted academic writing from three dimensions: impact, probability, and mitigation cost, thereby identifying high-risk factors.

**Results:**

The findings reveal that AI-assisted writing challenges fundamental principles of traditional copyright law, with judicial practice tending to position AI as a creative tool while emphasizing human agency. Regarding academic integrity, new risks, such as “credibility illusion” and “implicit plagiarism,” have become prominent in AI-generated content, necessitating adaptive regulatory mechanisms. Research data protection and personal information security face dual challenges in data security that require technological and institutional innovations.

**Discussion:**

Based on these findings, we propose a three-dimensional regulatory framework of “transparency, accountability, technical support” and present systematic policy recommendations from institutional design, organizational structure, and international cooperation perspectives. The research results deepen understanding of legal attributes of AI creation, promote theoretical innovation in digital era copyright and academic ethics, and provide practical guidance for academic institutions in formulating AI usage policies.

## Graphical abstract

This graphical abstract succinctly presents the key issues examined in this study as well as the main analytical framework. This illustration presents a legal regulatory framework and risk assessment for AI-assisted academic writing. At its center lies the core concept of AI-assisted academic writing, with icons symbolizing cognitive processing and document creation, representing the integration of artificial intelligence and academic writing. The main framework comprises three dimensions, shown as interconnected circular segments. The blue section represents academic integrity, emphasizing critical elements of transparent usage and accountability in AI applications; the orange section indicates data security, highlighting the implementation of privacy protection and security measures; the green section addresses copyright protection, focusing on ownership recognition and infringement prevention of AI-generated content. The lower right shows the risk assessment matrix that quantifies risk factors through three dimensions: impact, probability, and mitigation cost, with high-risk factors identified as “human creation” requirements, hallucination detection, the accuracy of content, and leakage of research results.

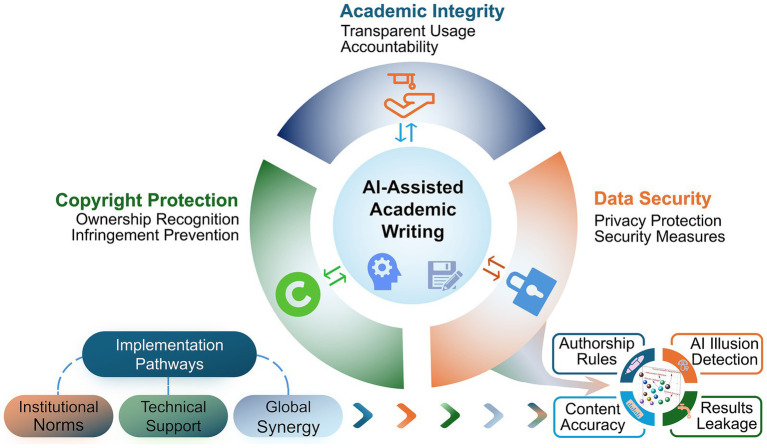

## Highlights

- Comprehensive legal framework for AI-assisted academic writing, integrating copyright protection, academic integrity, and data security dimensions by analyzing judicial practices, institutional policies, and emerging cases.
- Courts tend to position AI as a creative tool while emphasizing human agency, suggesting a pragmatic approach to resolving copyright disputes in AI-assisted academic writing.
- The analysis of guidelines from publishers and institutions reveals new risks associated with AI-assisted writing, including “credibility illusion,” implicit plagiarism, and weakening critical thinking skills.
- A three-dimensional regulatory framework (transparency, accountability, and technical support) is proposed, providing actionable guidance for institutions to develop AI usage policies.
- The study synthesizes data from multiple sources, including court decisions, institutional policies of elite universities, and international regulatory frameworks, offering evidence-based recommendations for legal regulation.
- Constructed a risk assessment matrix that quantifies risks through three dimensions (impact, probability, and cost), visualizing the magnitude of comprehensive risks.
- The lower left shows three implementation pathways: institutional norms establish regulatory guidance, technical support ensures the safe use of AI tools, and global synergy promotes consensus among academic communities. Bidirectional arrows indicate dynamic interactions. The graduation cap represents academic integrity, the lock symbol represents security measures, and the copyright symbol represents intellectual property. The pen represents creation, the mushroom represents hallucination, the ruler represents precision, and the tap represents leakage. These elements demonstrate the relevant legal, ethical, and technical environment, as well as high-risk factors that require prior attention, which together with the implementation pathways, establish a balanced and sustainable legal regulation for AI-assisted academic writing.

## 1 Introduction

The rapid development of artificial intelligence technology is profoundly reshaping the paradigm of academic writing. With their exceptional natural language processing capabilities, large language models (LLMs) represented by GPT and Claude have become indispensable auxiliary tools in academic writing (Meyer et al., 2023). These tools help review the literature, provide writing suggestions, optimize expression, and participate in multiple stages of the creative process (ElSayary, 2023; Kostka and Toncelli, 2023). However, the widespread application of AI writing assistance tools has also brought about several pressing legal issues. In particular, traditional legal norms and academic rules face unprecedented challenges in copyright attribution, academic integrity maintenance, and data security protection (Liebrenz et al., 2023; Abbott, 2020).

The academic community has extensively discussed the legal implications of AI-assisted writing. In the realm of copyright, scholars have developed diverse theoretical perspectives on issues such as the attribution of AI-generated content and the legal characterization of collaborative creation (Guadamuz, 2017; Oratz et al., 2024). Regarding academic integrity, major journals, and research institutions have successively issued AI usage guidelines, trying to balance technological innovation and academic standards (COPE, 2023; Gulumbe et al., 2024). In the field of data security, as countries continue to improve their data protection legislation, increasing attention has been paid to issues such as the legitimacy of AI training data and user privacy protection (Xinying et al., 2023; Xiao, 2024). However, research has been limited to specific domains, needing a systematic legal analysis framework (Moya et al., 2024).

**Figure 1:**
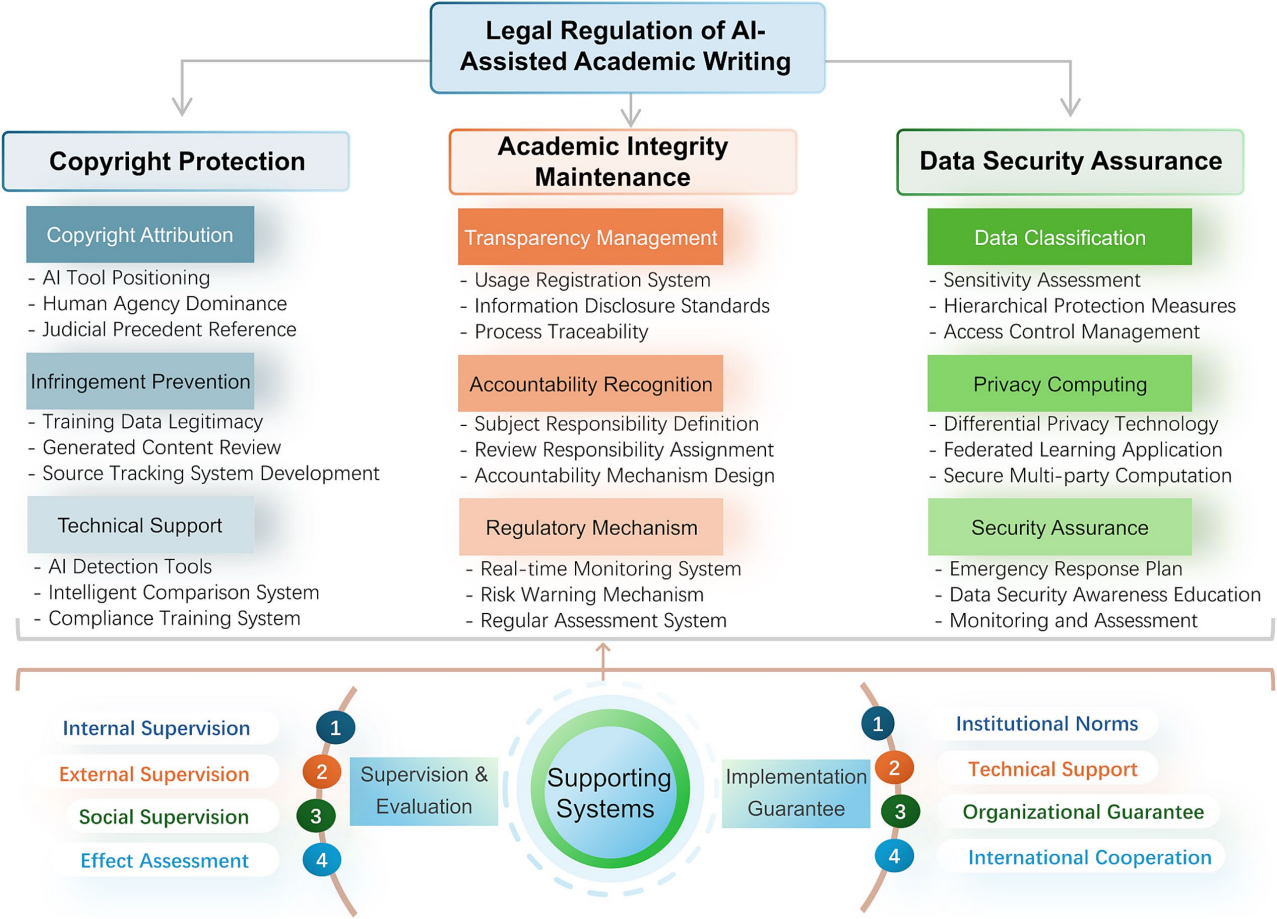
Comprehensive legal regulatory framework for AI-assisted academic writing. This figure corresponds to the three primary issues and main strategies discussed in Sections 2, 3, 4, and 6, aiming to provide a concise visual overview for readers. The overall layout has been designed with visual balance in mind, maintaining logical consistency while avoiding redundancy.

This study constructs a comprehensive legal regulatory framework encompassing copyright protection, academic integrity maintenance, and data security assurance (Figure 1). Through analyzing relevant legal norms, typical cases, and policy documents, combined with observations of AI-assisted writing practices, we explore the legal issues involved and their potential solutions. Theoretically, this research aimed to deepen the understanding of the legal attributes of AI creation, improve copyright theory in the digital age, and promote innovation in academic ethics theory. At the practical level, it provides references for developing AI usage policies, helping regulate academic writing behavior and prevent legal risks.

The significance of this research lies in (i) systematically reviewing and analyzing core legal issues in AI-assisted academic writing, including copyright protection, academic integrity maintenance, and data security assurance to construct a complete analytical framework; (ii) comparing relevant domestic and international policies and practical cases to summarize the main legal risks of AI-assisted academic writing and their preventive measures, providing practical guidance; and (iii) offering forward-looking suggestions for formulating AI usage policies and regulations based on existing experience and theoretical exploration.

As AI technology continues to evolve, academic writing will undergo constant transformation (Kim et al., 2024). The balance between promoting technological innovation and maintaining academic standards remains an important topic that requires continued exploration by the academic community. This systematic investigation of legal issues in AI-assisted academic writing will contribute to building a favorable academic ecosystem for the future.

## 2 Copyright issues

As a new creative paradigm, AI-assisted academic writing profoundly challenges the fundamental concepts and institutional framework of traditional copyright law. With the widespread application of large language models such as ChatGPT in academic writing, essential legal issues such as copyright attribution, originality determination, and infringement assessment require urgent clarification (Abbott, 2020). This chapter systematically explores copyright attribution disputes and infringement risk prevention in AI-assisted academic writing from both theoretical analysis and practical response perspectives.

### 2.1 Copyright attribution disputes

Copyright attribution in AI-assisted creation has become a focal point of academic controversy. Traditional copyright law emphasizes “human creation” as an essential requirement, highlighting that works should reflect the original expression (Guadamuz, 2017). However, in AI-assisted writing scenarios, the creative process often involves complex human–machine interaction, making the boundaries of originality increasingly ambiguous. Three main theoretical perspectives have emerged in academia: AI tool theory, AI independent creation theory, and collaborative creation theory (Xiao, 2024), each interpreting the copyright attribution of AI-generated content from different angles.

The AI tool theory advocates positioning AI systems as auxiliary creative tools, with the copyright of their generated content belonging to the users. This perspective has gained widespread support in judicial practice. For example, in the “Shenzhen Tencent v. Yingweinuo case” (Dreamwriter case), the court determined that AI-generated financial reports possess originality and that their copyright should belong to the entity that organized and guided the creative process (Abbott, 2020). This case provides an essential reference for copyright recognition in AI-assisted academic writing, emphasizing the dominant position of human subjects in the creative process.

However, the US Copyright Office has taken a more conservative stance toward AI creation. In Thaler v. In the US Copyright Office case, the court explicitly rejected the copyright registration application listing an AI system as an author, reaffirming the fundamental status of the “human creation” requirement (Oratz et al., 2024). This position has had profound implications in academic publishing, with mainstream academic journals generally requiring authors to assume full responsibility for AI-assisted created content while explicitly prohibiting the listing of AI tools as authors (COPE, 2023).

### 2.2 Infringement risk prevention

Copyright infringement risks in AI-assisted academic writing stem primarily from the legality of AI system training data and the infringement risks of generated content. Regarding training data, large language models use large amounts of potentially copyright-protected textual materials during training, and the legal nature of this usage remains controversial (European-Union, 2019). The Authors Guild v. OpenAI case centrally reflects this issue, and its judgment will significantly impact the legitimate scope of AI training data usage (Niu, 2023).

Regarding generated content, AI systems may tend to reproduce expression patterns from training data, increasing the potential risks of copyright infringement (Xiao, 2024; European-Union, 2019). Research indicates that large language models may unconsciously reproduce (copy or adapt) expressions from training data during generation, and this “latent derivation,” where AI systems subtly incorporate preexisting expressions without explicit attribution, presents new challenges for copyright protection.

In response to these risks, we propose systematic prevention mechanisms. First, we recommend improving AI usage registration systems that require researchers to maintain detailed records of AI tool usage, including key information such as purpose, scope, and extent. Second, we should develop specialized content similarity checks and source tracing systems to identify potential infringement issues quickly through technical means. Finally, we should establish clear review standards to regulate the limits of AI-assisted creation (Gulumbe et al., 2024).

### 2.3 Technical infrastructure and implementation framework

#### 2.3.1 AI content detection tools

Currently, multiple detection tools are available to identify AI-generated text. Representative detection tools include ZeroGPT, developed specifically to identify AI-generated content and accurately distinguish between AI-generated and human-written texts. Based on DeepAnalyse technology and a training corpus of more than 10 million articles, this tool achieves high accuracy while maintaining a low false-positive rate (Liu et al., 2024). Copyleaks, which focuses on plagiarism detection, has added functionality to identify AI-generated content. It uses machine learning algorithms to detect text sources, creation methods, and similarity, helping identify latent derivations during the review process (Mouli et al., 2024; Martins et al., 2024). Turnitin’s AI detection module is integrated into its anti-plagiarism system, capable of identifying AI-generated content while detecting plagiarism (Baron, 2024). This tool facilitates using a unified platform for multiple detection purposes, reducing operational costs.

These specialized tools can quickly filter content and provide detailed reports, helping identify possible academic misconduct. While commercial software generally performs better, tool selection should be based on the type of content analyzed (Martins et al., 2024), and regular reviews and updates are necessary to ensure effectiveness.

#### 2.3.2 Text source tracing and intelligent comparison

To address issues of latent derivation, we propose establishing intelligent text source tracing and comparison systems to help track text origins and evaluate originality levels. ① Text source database: establish an academic text source database to enable precise tracking of content origins by recording sources and usage records. This database can also integrate text comparison systems to identify whether AI-generated content is excessively derived from training data. ② Intelligent comparison system: AI-assisted intelligent comparison systems can conduct detailed comparisons between generated content and existing academic works to evaluate their originality levels. These systems can effectively distinguish between original and derivative content using deep learning models, helping academic institutions strengthen the oversight of AI-generated content.

#### 2.3.3 Overview of data security assurance

Data security is also crucial for the compliant use of AI-assisted writing within the technical support system. For example, privacy protection technologies such as differential privacy and federated learning can, to some extent, ensure data security. For a detailed discussion of the comprehensive privacy protection system, refer to “Technical Aspects” under “Security Mechanism Innovation Development” in Chapter 4, “Data Security and Privacy Protection.”

#### 2.3.4 Universal education and compliance awareness development

Beyond technical tool support, strengthening copyright education is equally important. Through systematic training, users’ copyright awareness and ability to use AI tools compliantly can be effectively enhanced. This education should not be limited to notification but should help users fully understand legal risks in AI-assisted creation and master effective preventive measures. For example, academic institutions can regularly organize AI tool usage training, copyright compliance lectures, and case analysis sessions to improve awareness of legality and responsibility in academic writing (Dabis and Csáki, 2024). The combination of educational and technical approaches helps establish a sound technical support system and forms dual protective measures of “technology and education,” enabling us to avoid copyright risks while using AI technology effectively.

#### 2.3.5 Coordination and development of international rules

Given the transboundary nature of AI technology, strengthening the coordination of international copyright rules has become particularly important. Currently, there are significant differences among countries regarding copyright protection for AI-created works. Some countries adopt a relatively open attitude, recognizing copyright protection for AI-assisted creations, while others maintain a more cautious stance, emphasizing the central role of human creation. These differences affect cross-border academic collaboration and present new challenges for copyright protection.

### 2.4 Summary

Copyright issues in AI-assisted academic writing involve balancing the interests of multiple legal entities, and its standardized development requires consensus across multiple dimensions. Current legal practice generally tends to position AI as a creative tool, highlighting the leading role of humans in the creative process and providing a basic framework for building copyright protection systems for AI-assisted academic writing (Gaffar and Albarashdi, 2024; Meyer et al., 2023). As AI technology continues to evolve, relevant legal rules still need constant adjustment and refinement. This refinement should proceed simultaneously on three levels: (i) at the international level, promote the coordination and unification of copyright rules to construct a protection framework adapted to the characteristics of the AI era; (ii) at the practical level, seek a balance point between innovative protection and standardized use to ensure coordination between technological development and legal protection; (iii) at the institutional design level, maintain the openness and flexibility of rules by establishing dynamic adjustment mechanisms that allow legal norms to respond promptly to new challenges brought by technological changes.

## 3 Academic integrity

With the deep integration of AI technology into academic writing, traditional academic integrity norms face unprecedented challenges. Academic integrity, as a core value of the academic community, not only reflects the adhesion to fundamental ethical principles, such as courage, fairness, honesty, responsibility, respect, and trust (Moya et al., 2024), but also requires new interpretations in the context of technological transformation. This chapter delves into the novel challenges and regulatory pathways for academic integrity in the era of artificial intelligence.

### 3.1 Emerging forms of academic misconduct

Academic misconduct involving AI-assisted writing exhibits distinct contemporary characteristics. Compared to traditional plagiarism and copying behaviors, these new forms of academic misconduct are more covert and complex (Liebrenz et al., 2023). Three typical manifestations can be identified: (i) undisclosed use—using AI tools to generate content without explicit declaration, obscuring the true creative process; (ii) over-reliance—transforming AI tools from auxiliary means to substitutive tools, weakening researchers’ independent thinking; (iii) data manipulation—using AI technology to manipulate research data or fabricate false references, jeopardizing the authenticity of academic outcomes (De Angelis et al., 2023).

Of particular concern is the “credibility illusion” problem in AI-generated content, arising from ability of AI system to generate seemingly rigorous but potentially biased or erroneous content, leading readers to develop misplaced trust. This “hallucination” phenomenon in AI not only affects the reliability of academic output (Liebrenz et al., 2023; De Angelis et al., 2023; Waldo and Boussard, 2024) but also increases the difficulty in identifying academic misconduct (Meyer et al., 2023). Previously, nearly half of the audiences were concerned by some form of online misinformation (Saltz et al., 2021); currently, some scholars, fearful of potential of AI for error, strongly advocate against its use in academic writing (Emsley, 2023).

### 3.2 Construction of new academic integrity standards

In response to these challenges, the international academic community is actively constructing new standards of academic integrity adapted to the AI era. Leading journals such as *Nature* and *Science* have initiated the publication of AI usage guidelines, providing a crucial reference for regulating AI tools in academic writing. These guidelines generally emphasize two core requirements: (i) the principle of transparency, requiring authors to disclose the usage of AI tools usage explicitly; (ii) the principle of accountability, clearly stating that the authors bear full responsibility for the accuracy of AI-generated content (Gulumbe et al., 2024).

The Statement of the Committee on Publication Ethics (COPE) further clarifies the positioning of AI tools, emphasizing that AI systems do not possess independent legal personhood and cannot be regarded as authors. This positioning provides important guidance to delineate the boundaries of responsibility in human–machine collaboration (COPE, 2023). The European Research Council (ERC), from a funding perspective, emphasizes researchers’ “complete and sole authorial responsibility” for AI-assisted generated content (Moller-Nielsen, 2023), further strengthening academic accountability awareness.

The exploration of AI usage policies by world-class universities is equally worthy of reference. Harvard University Information Technology (2024), Massachusetts Institute of Technology (2024), Stanford University (2023), and Washington State University (2023) have successively provided guidance on the use of generative AI, forming a basic consensus of “responsible use, emphasis on safety, maintaining transparency, and upholding academic integrity and ethical regulations.” The evolutionary trajectory of these policies indicates that the attitudes of higher education institutions toward AI technology are gradually transitioning from initial caution and restriction to openness and active regulation (Dabis and Csáki, 2024). These explorations provide valuable experience in constructing academic integrity systems in the new era.

### 3.3 Construction of regulatory mechanisms

Establishing effective regulatory mechanisms is the key to maintaining academic integrity. Based on an analysis of the literature, this study proposes a three-dimensional regulatory framework of “transparency, accountability, and technical support.” In the transparency dimension, an AI usage registration system is required to ensure the traceability of the creative process. This includes not only disclosure of the usage of AI tools but also descriptions of specific usage scenarios and the extent of enhancing academic output transparency. In the accountability dimension, the focus is on clearly defining responsibility boundaries and establishing violation-handling mechanisms. Through the establishment of comprehensive supervision mechanisms, practical responsibility constraints are formed. Developing AI content detection tools and building intelligent monitoring and early warning systems is necessary in the technical support dimension. These technical means can help identify potential academic misconduct and provide an objective basis for evaluating academic output quality.

### 3.4 Capacity building in academic communities

Maintaining academic integrity requires institutional norms and strengthening capacity building within academic communities. Incorporating AI technology into teaching practices by designing comparative analysis tasks and conducting AI output questioning training can effectively enhance users’ critical thinking abilities. This training model helps users better understand the limitations of AI tools and strengthens their ability to distinguish between true and false information.

Meanwhile, continuous education in academic ethics is an indispensable component. This education should go beyond the notification of traditional norms to help academic communities fully understand the new implications of academic integrity in the era and cultivate responsible usage habits (Dabis and Csáki, 2024). Positive academic ethical practices can be promoted through various educational forms, such as case analysis and sharing experiences.

### 3.5 Summary

Although AI-assisted academic writing provides new possibilities for knowledge innovation, it also challenges academic integrity. Constructing adaptive regulatory mechanisms requires finding an appropriate balance between encouraging innovation and maintaining integrity. Future research should focus on the long-term impact of AI applications, explore more effective regulatory models, and promote the healthy development of academic ecosystems (Ofosu-Ampong, 2024). In particular, regulatory frameworks must maintain sufficient flexibility to adapt to new application scenarios and ethical challenges in rapid technological iteration. A favorable ecosystem that promotes technological innovation and maintains academic integrity can be built through the collaborative efforts of stakeholders.

## 4 Data security

Privacy and data security issues have become increasingly prominent in AI-assisted academic writing. Ensuring data security while promoting innovation in AI technology has become a significant challenge facing academic institutions (European-Union, 2019).

### 4.1 Data security risk identification

Data security risks in AI-assisted academic writing manifest primarily in research data protection and personal information security. The core risks at the research data level stem from the possible leakage of critical academic assets such as unpublished research findings, original experimental data, and innovative research methods. This leakage risk occurs through two pathways: (i) the “memory effect” of AI models (Xinying et al., 2023), where sensitive information is retained during training and potentially leaked in subsequent outputs; (ii) security vulnerabilities in data transmission, where important research data face risks of unauthorized access.

In terms of protecting personal information, risks present more complex characteristics. When AI systems process academic texts, they may involve authors’ personal information and privacy data related to research subjects and collaborators. Of particular concern is that large language models, through deep learning and associative analysis, can infer user identity characteristics and behavioral patterns from seemingly unrelated data (Kathrin, 2024). Although this capability enhances service personalization, it also poses serious privacy leakage risks.

### 4.2 Construction of legal protection systems

Faced with increasingly complex data security challenges, the international community has gradually constructed a multilayered legal protection system. The European Union’s General Data Protection Regulation (GDPR) establishes six strict principles for personal data processing, with data minimization and purpose limitation principles being particularly important, setting stringent standards for personal data processing. These regulations require data processing to follow the principles of adequacy, relevance, and necessity (GDPR-INFO-EU, 2018), providing important guidance to academic institutions to standardize their data processing procedures.

The California Consumer Privacy Act (CCPA) of the United States focuses on rights protection, clearly defining data subjects’ rights to know, delete, and opt-out. This legislative model strengthens control over personal data while providing an important reference for academic institutions handling personal information (State of California Department of Justice, 2024). Meanwhile, Cybersecurity Law and Personal Information Protection Law of China have built a comprehensive data protection framework (Xinying et al., 2023); these laws require data processors to adopt the necessary measures to protect data security and clearly stipulate the basic principles for personal information processing.

### 4.3 Innovation and development of security mechanisms

Effective data security protection mechanisms require dual support from technology and management innovation.

#### 4.3.1 Technical level

The new generation of privacy computing demonstrates broad application prospects through technologies such as encrypted transmission and access control. Differential privacy technology protects individual privacy while ensuring data analysis accuracy by adding random noise to the data. This technology is suitable for protecting sensitive data processing in AI-assisted academic writing, preventing data leakage. Federated learning achieves an innovative paradigm of “data non-disclosure, model sharing” through local data processing and collaborative model training, effectively reducing data leakage risks (Hartmann and Kairouz, 2023). This technology suits multi-institutional collaborative environments, helping protect participants’ data security in academic writing collaborative projects. Its advantages include data isolation, where participant’s data remain stored locally, and control rights, where participants can decide when and how to share information. The development of secure multiparty computation (MPC) technology, exemplified by the Stanford University MPC platform (Prabhakaran and Sahai, 2013), utilizes advanced cryptographic principles that allow multiple parties to perform secure computation and collaboration without exposing raw data. Microsoft and other institutions have applied MPC technology to validate AI models, enabling AI-assisted writing under privacy protection (Chandran et al., 2022; Jawad, 2023; Seo, 2021), and expanding application scenarios in academic writing.

#### 4.3.2 Management system level

*Data classification system establishment*: adopt differentiated protection measures for data of different security levels. This system can help academic institutions optimize resource allocation by focusing key security measures on highly sensitive data, thus improving overall data security management efficiency.

*Emergency response mechanism establishment*: academic institutions should establish comprehensive emergency response mechanisms to respond to data security incidents rapidly. By establishing dedicated response teams and processes, quick action can be taken when data breaches or security incidents occur, effectively controlling risks. For example, organizations should develop targeted data breach contingency plans and response processes, and conduct regular emergency drills to improve practical response capabilities.

*Data security awareness education and compliance habit cultivation*: compliance and security education are crucial in data management. As mentioned above, multiple renowned universities and institutions have begun formulating guidelines and regulations for AI-assisted writing, clarifying responsibilities, and strengthening security awareness training for researchers and students. Academic institutions are advised to conduct regular data security training to enhance personnel’s security awareness and ability to use AI tools compliantly, thereby forming an all-staff participatory data security culture.

### 4.4 Practical recommendations

Based on a comprehensive understanding of the characteristics of AI-assisted academic writing, we propose the following optimization recommendations that align with our previously discussed framework: (i) establish an admission evaluation system for AI tools, emphasizing their compliance and security in data processing. This evaluation should integrate with the oversight mechanisms discussed in Chapter 3 while adhering to the copyright principles established in Chapter 2. (ii) Improve data desensitization mechanisms to ensure adequate protection of sensitive information when using AI tools. This approach should sync with our proposed three-dimensional regulatory framework to create a comprehensive protection system. (iii) Implement real-time monitoring systems to identify and address potential data security vulnerabilities quickly. This system should complement the technical infrastructure outlined in previous sections while supporting broader regulatory objectives.

### 4.5 Summary

Data security risks are constantly evolving as AI technology continues its rapid evolution. Academic institutions must remain vigilant and continuously update security strategies to adapt to new challenges. Key focus areas include the development of security assessment standards for AI systems, implementing emerging privacy protection technologies, and standardizing cross-border data flow management. Through the continuous refinement of technical measures and optimization of management processes, a comprehensive data security protection system can be established, providing solid safeguards for the healthy development of AI-assisted academic writing.

The recommendations align with the broader framework presented in this document, creating a cohesive approach to addressing the interconnected challenges of copyright protection, academic integrity, and data security in the AI era.

## 5 Risk factor matrix visualization

Based on our systematic analysis of copyright protection, academic integrity maintenance, and data security assurance in AI-assisted academic writing, we have constructed a risk assessment matrix model by categorizing, coding, and quantifying all identified risks. This matrix evaluates risks in three dimensions: impact, probability, and mitigation cost, utilizing a comprehensive approach to risk assessment. The key evaluation criteria include the following:

Impact assessment considers the following: overall influence on the academic community, damage to institutional reputation, effects on researchers, and severity of legal consequences.

Probability evaluation is based on existing research reports, judiciary case studies, institutional feedback, and technical characteristics.

Cost calculation accounts for required technological investment, human resource demands, time expenditure, and institutional framework development costs.

We employed a multi-step iterative process to determine the score of each risk factor in the three dimensions mentioned above. First, conduct a systematic literature review (covering court precedents, academic integrity guidelines, and institutional policies) to identify potentially relevant risk events and collect reference information on their occurrence probabilities or severity.

Subsequently, 15 experts (legal scholars, AI researchers, and institutional managers) were consulted, who independently proposed initial scores on a scale of 1–10 for each dimension. Afterward, we compiled and anonymized these scores, holding group discussions to address any significant discrepancies. Where substantial disagreements arose, we applied the median or majority-vote approach as appropriate.

The third step is to calibrate the final score using quantitative indicators from relevant literature. Ensure that the numerical allocation of each dimension reflects both expert judgment and verifiable indicators.

**Figure 2:**
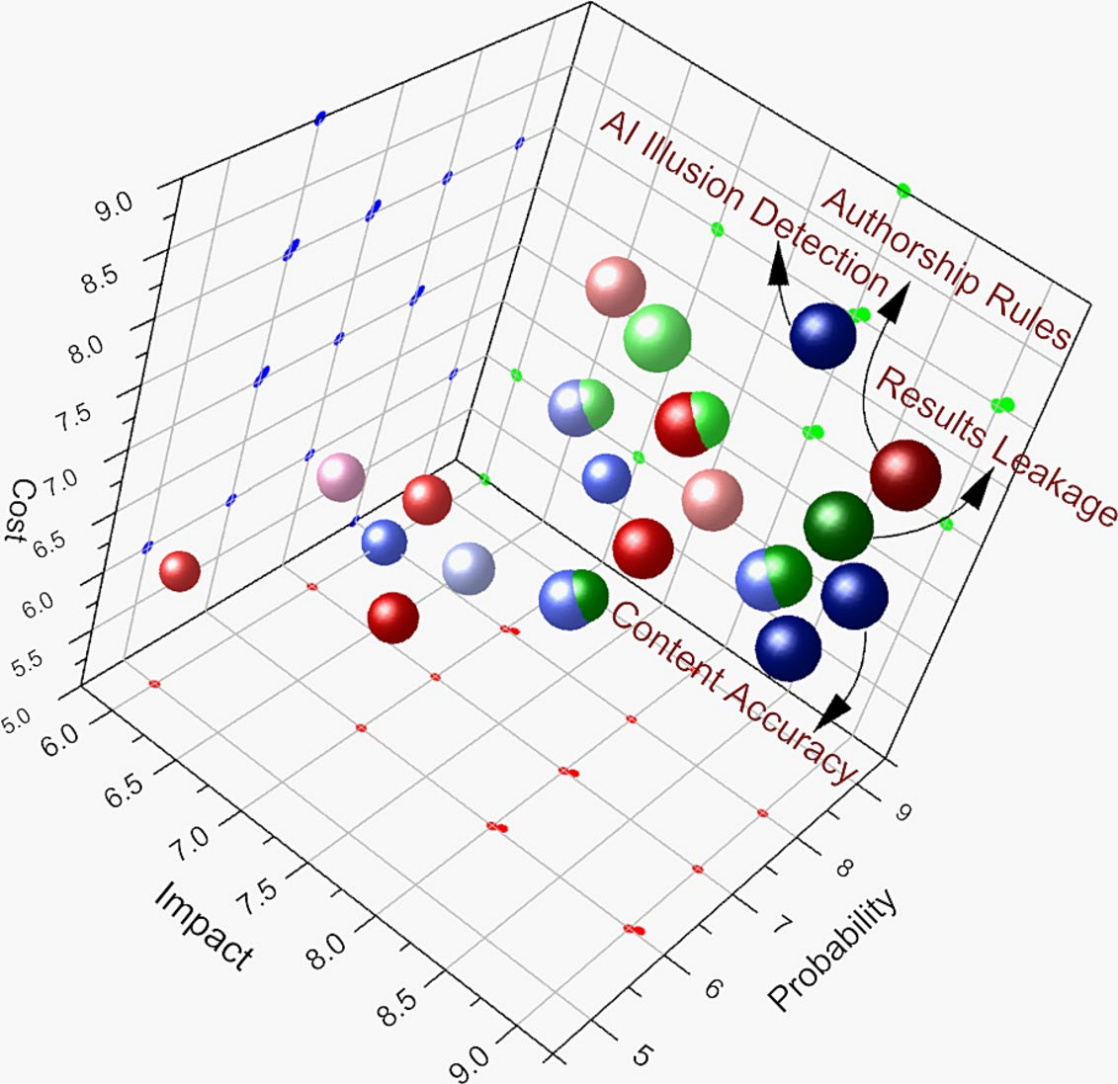
Risk assessment matrix for AI-assisted academic writing. The three-dimensional scatter plot visualizes comprehensive risk assessment results across the dimensions of copyright protection (blue spheres), academic integrity (green spheres), and data security (red spheres). The use of different shades or hues of the same color reflects the relative high or low comprehensive risk value (CR) among similar risks. Risk factors are evaluated through three metrics: impact (x-axis, 1–10), probability (y-axis, 1–10), and mitigation cost (z-axis, 1–10). The size represents the comprehensive risk value (CR), calculated using weighted factors. Impact (0.45), probability (0.35), and cost (0.20). Four high-risk factors (CR ≥ 8.0) were identified: traditional “human creation” requirements (A1.1, CR 8.45), “hallucination” detection (B1.2, CR 8.35), content accuracy (B1.1, CR 8.25), and research results leakage (C1.1, CR 8.05). The clustering of these factors in the upper region highlights critical areas that require immediate attention in strategic planning and resource allocation. For clarity of visualization, coordinate adjustments (+0.05 in all three dimensions) were applied to four risk factors with identical assessment values: raw data leakage (C1.2), methods leakage (C1.3), subject rights (C2.1) and model memory effect (C2.2), while maintaining the integrity of the original risk assessment values.

The comprehensive risk value (CR) is then calculated by weighting the allocation of these three risk factors. Weighting considerations include target constraints, risk tolerance, industry characteristics, and empirical data. Through team discussions, a review of the literature, and expert consultation (Vose, 2008; Saaty, 1980), the following weights were determined: impact (0.45), probability (0.35), and cost (0.20). The corresponding data from Table 1 were visualized using Origin 2025 to create a 3D scatter plot (Liang et al., 2024) that clearly illustrates which risk factors (top-right section in Figure 2) should be prioritized among multiple factors, with the size of each sphere representing its comprehensive risk value (Figure 2).

**Table 1:** Assessment of risk factors for AI-assisted academic writing.

Code	Risk factors	I	P	C	CR	S	Remarks
A1.1	Authorship rules	9	8	8	8.45	Largest	Legal principle challenges
A1.2	Human–AI collaboration	8	7	7	7.45		Common judicial issues
A1.3	AI tool positioning	7	6	6	6.45	Third smallest	Policy clarification needed
A2.1	Training data copyright	8	7	8	7.65		Frequent litigation
A2.2	Usage authorization	7	6	7	6.65		Complex agreements needed
A2.3	Legal sources	6	5	6	5.65	Smallest	Strict review required
A3.1	Data expression reuse	8	8	7	7.75		Hard to identify and prove
A3.2	Traceability issues	7	8	8	7.55		Technical complexity
A3.3	Expression repetition	6	7	6	6.35	Second smallest	Comparative analysis needed
B1.1	Content accuracy	9	8	7	8.25	Third largest	Impact on credibility
B1.2	AI illusion detection	8	9	8	8.35	Second largest	Detection challenges
B1.3	Error propagation	9	7	7	7.90		Broad impact
B2.1	Undisclosed use	7	9	6	7.50		Common issue
B2.2	Over-reliance	6	8	5	6.45	Third smallest	Long-term guidance needed
B2.3	Data manipulation	9	6	8	7.75		Academic norm violation
B2.4	False citations	8	6	7	7.15		Strict review needed
B3.1	Critical thinking	7	8	7	7.35		Training required
B3.2	Originality issues	8	7	7	7.40		Core academic values
B3.3	Research depth	7	7	6	6.75		Quality control
C1.1	Results leakage	9	7	8	8.05	Fourth largest	Core competency risks
C1.2	Raw data leakage	8	6	7	7.05		Data protection
C1.3	Methods leakage	9	6	8	7.75		Innovation protection
C2.1	Subject rights	8	7	8	7.65		Compliance requirements
C2.2	Model memory effect	7	8	7	7.35		Technical barriers
C2.3	Cross-border flow	8	6	9	7.55		Cross-border compliance

A1.1-A3.3, risks related to copyright; B1.1-B3.3, risks related to academic integrity; C1.1-C2.3, risks related to data security. I, Impact; P, probability; C, cost of mitigation; S, size of the sphere (indicating risk magnitude); CR, comprehensive risk value, calculated as CR = 0.45 × I + 0.35 × P + 0.20 × C. In this weighting system, the impact (I), directly associated with the severity of the potential loss, is assigned the highest weight (0.4–0.6). Probability (P), representing the probability of occurrence of risk, is also a significant factor with moderate weight (0.2–0.4). Although the mitigation cost (C) is important, it is comparatively less impactful, thus allowing it to be assigned a lower weight (0.1–0.3).

Interpretation of matrix results: in the three-dimensional risk matrix visualization, risk factors are differentiated by color-coded spheres: The blue spheres represent copyright risks (Category A), the green spheres represent academic integrity risks (Category B), and the red spheres represent data security risks (Category C).

The matrix reveals four prominent high-risk factors (CR ≥ 8.0) clustered in the upper left region of the figure: A1.1: challenge to traditional “human creation” requirements (CR 8.45); B1.2: “hallucination” detection (CR 8.35); B1.1: content accuracy (CR 8.25); C1.1: unpublished research results leakage (CR 8.05).

These high-risk factors share common characteristics: high impact scores (8–9 points), high probability ratings (7–9 points), and substantial mitigation costs (7–8 points).

Clustering these risks in the matrix’s upper region underscores their critical nature, demanding priority attention in strategic planning and resource allocation. This visualization effectively illustrates the interconnected nature of legal, ethical, and security challenges in AI-assisted academic writing, providing a quantitative basis for developing targeted intervention strategies.

Drawing on the quantitative risk assessment framework outlined above and synthesizing our comprehensive analysis in previous chapters, we propose a series of evidence-based policy recommendations.

## 6 Recommendations and prospects

Effective regulation and governance will promote public trust in AI-assisted generated content (OECD, 2023). Given the rapid advancement of artificial intelligence technology, constructing a scientifically sound policy framework to achieve a balance between technological innovation and regulatory management has become a crucial challenge facing academia. We present the following actionable, staged policy recommendations based on our previous analysis of core issues in copyright protection, academic integrity maintenance, and data security assurance, particularly the high-risk factors identified in our risk matrix assessment.

### 6.1 Systematic construction of institutional framework

AI management policies of academic institutions generally exhibit fragmented and reactive characteristics (Dabis and Csáki, 2024), necessitating the establishment of a systematic governance framework. Research indicates that an effective AI governance framework should adhere to three fundamental principles: legitimacy (Erman and Furendal, 2022), operability (GAN-Integrity, 2024), and dynamic adjustment (Appiah, 2024). Therefore, we recommend improving the management system from the perspectives of institutional design and organizational structure.

A multilevel management system should be constructed at the institutional design level, including basic usage regulations, specific operational guidelines, and supporting supervision mechanisms (Harvard University Information Technology, 2024). Experience shows that embedding AI tools within existing academic management frameworks can reduce policy implementation resistance and improve execution effectiveness (Massachusetts Institute of Technology, 2024; Panthagani and Zevallos, 2024). In particular, clear standards and procedures must be established in copyright protection and academic integrity maintenance to ensure the policy is operable.

At the organizational structure level, a systematic management philosophy is required to construct collaborative mechanisms that coordinate stakeholder cooperation for the responsible application of AI application (OECD, 2023; Gulumbe et al., 2024). By establishing AI management committees, institutions can oversee policy formulation, technology assessment, and risk monitoring. The Stanford Graduate School initiative’s cross-disciplinary cooperation model is a valuable reference (Stanford-Seed-Funding, 2024), effectively bridging research, innovation, practice, and policy by promoting deep exchanges between scholars and external partners. This collaborative approach not only helps enhance the security and reliability of AI applications and ensures the standardized use of AI tools but also strengthens public trust in AI technology.

### 6.2 Specific implementation strategies

Taking into account the characteristics of AI-assisted academic writing, we propose a phased implementation strategy. The main task for short-term strategies is establishing registration systems and information disclosure mechanisms. Disclosure of AI tools can significantly enhance academic outputs, with transparency requirements that include code visibility, model decision processes and rationales, and social impacts (Lawton, 2024). Meanwhile, training in AI literacy (skills, knowledge, and understanding of opportunities and risks) helps improve compliance with AI tool usage (Ng et al., 2021). Users with greater AI literacy typically demonstrate more cautious approaches to AI-generated content and greater awareness of compliance awareness (Kühl et al., 2024). Long-term strategies focus on constructing intelligent regulatory platforms and implementing advanced detection technologies to achieve early detection and monitoring of AI usage. Furthermore, dynamic evaluation, regular evaluation, and timely optimization of management measures are essential to ensure the continued effectiveness of the policy framework.

### 6.3 Technical support and assurance

Effective policy implementation requires robust technical support: (i) develop integrated AI management platforms that allow full traceability of usage processes; (ii) construct intelligent evaluation systems to improve management efficiency through data analysis; and (iii) perfect security protection mechanisms to ensure system stability and data security.

### 6.4 International cooperation and standardization

Given inherent characteristics of AI technology, strengthening international cooperation and standardization becomes particularly important. International organizations, represented by UNESCO, promote the development of global ethical standards for AI applications in education (UNESCO, 2023). Practical considerations include the following: (i) actively participate in an international standard setting to promote unified AI usage norms; (ii) establish coordination mechanisms for cross-border data flow to ensure data security and compliant use; and (iii) strengthen international exchange and sharing of experience to promote best practices.

### 6.5 Prospects and technology dependency risks

#### 6.5.1 Prospects

The development trends in AI-assisted academic writing are expected to manifest itself in the following aspects: at the technical level, the capabilities of the AI model will continue to advance, with human–machine collaboration becoming more sophisticated (Meyer et al., 2023); at the application level, personalization and intelligent features will become more pronounced; at the regulatory level, AI-based governance tools will see widespread adoption. Meanwhile, technology dependency, the digital divide, and ethical boundaries require increased attention.

Addressing these challenges requires academic institutions to maintain an open and innovative attitude while continuously refining policy frameworks through practice. In particular, in the context of rapid technological iteration, policymakers must possess a forward-looking vision, quickly grasp technological development trends, and ensure adaptability and effectiveness. Through continuous optimization and adjustment, we can promote the standardized application of AI technology in academia and facilitate a general improvement in academic research quality (Gulumbe et al., 2024).

#### 6.5.2 Technology dependency risks

Although AI tools provide writing support and data processing convenience, they may also negatively impact researchers’ independent thinking and critical analysis capabilities. Academic institutions should be vigilant about several long-term impacts:

*Critical thinking weakness*: in an AI-assisted writing environment, researchers may gradually become dependent on AI tools for content generation and literature analysis, reducing the frequency of independent thinking. This overreliance may weaken critical thinking, making researchers less capable of conducting in-depth studies of complex academic issues. Strategy: increase critical thinking and logical analysis courses to help researchers maintain independent judgment capabilities.

*Technical errors and “credibility illusion”*: AI tools may produce erroneous output due to data gaps or algorithmic bias. However, researchers might fail to detect these flaws, leading to the “credibility illusion.” Once incorporated into published academic work, such errors could seriously affect the quality of research and academic integrity. Strategy: establish AI-generated content review protocols, implementing human verification and validation to ensure content accuracy. Consider introducing content detection systems in academic institutions to help researchers examine the accuracy and compliance of generated content.

*Academic ethics issues*: with the popularization of AI-assisted writing, academia might gradually overlook existing academic moral and ethical norms. For example, researchers might cite AI-generated content without fully understanding its limitations or even directly claiming authorship. Strategy: emphasize the importance of academic ethics and establish clear AI usage guidelines that require full disclosure and proper citation when using AI tools. Additionally, we organize academic ethics and moral-themed lectures to enhance researchers’ sense of responsibility and moral self-discipline.

*Uncontrollability*: due to continuous AI technology iteration and upgrades, academia may struggle to fully control its development process, particularly regarding large language models’ dependency on training data and the speed of knowledge updates. This uncontrollability might lead academic research directions to be driven by technological development, gradually deviating from independent innovation paths. Strategy: conduct regular assessments of AI tools and establish clear usage boundaries to prevent excessive technological intervention in academic decisions. Consider forming oversight teams comprising AI experts, academic representatives, and ethics specialists to track AI technology development and ensure the balance between technology and academic research.

Technology dependency represents a key issue in the future development of AI-assisted writing. When seeking academic innovation, academic institutions must be vigilant about the negative impacts of technology dependency. Through establishing critical thinking training, perfecting AI content review mechanisms, strengthening academic ethics education, and setting technological usage boundaries, academia can achieve a balance between technological progress and academic independence, ensuring the standardization and sustainability of AI-assisted writing’s future development.

## 7 Conclusion

Artificial intelligence is reshaping academic writing paradigms with unprecedented speed and depth. This research, grounded in legal perspectives, has constructed a theoretical and practically valuable legal regulatory framework by systematically examining the core issues in AI-assisted academic writing, including copyright protection, academic integrity maintenance, and data security assurance. Our findings reveal the multidimensional challenges to traditional academic norms while providing insightful solutions to address these challenges.

In the domain of copyright protection, our research clarifies the challenges that AI-assisted writing poses to fundamental principles of traditional copyright law. Through an in-depth analysis of judicial practices, we find that mainstream jurisprudence positions AI as a creative tool while emphasizing human agency in the creative process. This positioning provides a basic framework for constructing copyright protection systems in AI-assisted academic writing and indicates directions for resolving ownership disputes in human–machine collaborative creation. Meanwhile, research demonstrates that effectively preventing infringement risks requires coordinated efforts across technical support, institutional norms, and educational training dimensions to build a multilayered protection system.

Regarding maintaining academic integrity, our research reveals new characteristics of academic misconduct in the AI era. In particular, the “hallucination” issue in AI-generated content tests traditional academic quality control mechanisms and poses new requirements for evaluating academic output reliability. The international academic community is actively constructing new academic norm systems adapted to AI era characteristics, maintaining academic writing credibility through strengthening transparency requirements and clarifying responsibility attribution. This provides a valuable reference for building a positive academic ecosystem.

Regarding data security assurance, our research thoroughly analyzes data leakage risks and personal information protection challenges in AI-assisted writing processes. Through a comparative study of various countries’ data protection laws, we reveal the advantages and limitations of current legal regulatory frameworks. Research indicates that establishing multilayered data security protection mechanisms is crucial to ensure the standardized development of AI-assisted writing. These findings not only enrich data law theory but also provide specific guidance for practical risk prevention.

The theoretical value of this research is threefold: (i) it deepens understanding of AI creation’s legal attributes, making innovative contributions to the copyright theory system in the digital age; (ii) it proposes new academic integrity standards aligned with AI era characteristics, promoting innovation in academic ethics theory; (iii) it constructs a comprehensive legal regulatory framework for AI-assisted academic writing, opening new theoretical perspectives for related research. At the practical level, it provides concrete guidance for academic institutions in formulating AI usage policies, particularly offering actionable recommendations in policy framework design, regulatory mechanism construction, and technical support systems.

Looking ahead, the continuous evolution of AI technology will further deepen its impact on academic writing. We recommend the following: (i) strengthening tracking research on the long-term effects of AI-assisted writing, particularly its profound impact on academic paradigm transformation; (ii) conducting broader cross-cultural comparative studies to explore characteristics and commonalities of AI applications under different academic traditions; (iii) investigating in depth how emerging technological developments impact and reconstruct academic writing norms.

In particular, in advancing the deep integration of AI technology with academic research, it is essential to maintain a legal orientation, striking a balance between embracing technological change and upholding the eternal values of academic research’s rigor and ethical compliance. This requires the evolution of legal norms, active participation, and rational consideration from the academic community. Only through these efforts can we ensure that AI technology truly serves the fundamental goals of academic research, promoting continuous innovation, and the development of human knowledge.

## Data Availability

The original contributions presented in the study are included in the article/supplementary material, further inquiries can be directed to the corresponding authors.
